# Enhanced Transmission of Drug-Resistant Parasites to Mosquitoes following Drug Treatment in Rodent Malaria

**DOI:** 10.1371/journal.pone.0037172

**Published:** 2012-06-06

**Authors:** Andrew S. Bell, Silvie Huijben, Krijn P. Paaijmans, Derek G. Sim, Brian H. K. Chan, William A. Nelson, Andrew F. Read

**Affiliations:** 1 Center for Infectious Disease Dynamics, Departments of Biology and Entomology, The Pennsylvania State University, University Park, Pennsylvania, United States of America; 2 Department of Biology, Queen’s University, Kingston, Ontario, Canada; 3 Fogarty International Center, National Institutes of Health, Bethesda, Maryland, United States of America; Kenya Medical Research Institute - Wellcome Trust Research Programme, Kenya

## Abstract

The evolution of drug resistant *Plasmodium* parasites is a major challenge to effective malaria control. In theory, competitive interactions between sensitive parasites and resistant parasites within infections are a major determinant of the rate at which parasite evolution undermines drug efficacy. Competitive suppression of resistant parasites in untreated hosts slows the spread of resistance; competitive release following treatment enhances it. Here we report that for the murine model *Plasmodium chabaudi*, co-infection with drug-sensitive parasites can prevent the transmission of initially rare resistant parasites to mosquitoes. Removal of drug-sensitive parasites following chemotherapy enabled resistant parasites to transmit to mosquitoes as successfully as sensitive parasites in the absence of treatment. We also show that the genetic composition of gametocyte populations in host venous blood accurately reflects the genetic composition of gametocytes taken up by mosquitoes. Our data demonstrate that, at least for this mouse model, aggressive chemotherapy leads to very effective transmission of highly resistant parasites that are present in an infection, the very parasites which undermine the long term efficacy of front-line drugs.

## Introduction

Global deployment of antimalarial drugs in the latter half of the 20^th^ century placed enormous selection pressure on human malaria parasites to evolve resistance. In many parts of the world, front-line drugs such as chloroquine and sulphadoxine-pyrimethamine (SP) are now ineffective against *Plasmodium falciparum*, and the available alternatives are increasingly threatened [1]–[3]. Indeed, the World Health Organization [4] considers the evolution of drug resistance by malaria parasites to be inevitable, and acknowledges that for as long as malaria is around, a drug-discovery pipeline will be required to replace drugs as they fail [5]. The speed of this ‘drug treadmill’ is determined primarily by the rate at which mutations conferring resistance arise and reach transmissible densities in an infection, and by the rate at which they spread in a population [6].

It has become apparent that malarial infections typically comprise multiple parasite genotypes, particularly in areas of high transmission (e.g. [7]–[18]). Within mixed infections, powerful genotype-genotype crowding effects can occur, as shown both by correlational epidemiologic evidence for human infections [9], [19]–[25] and by direct experimental investigation using the rodent malaria *Plasmodium chabaudi* in laboratory mice [26]–[35]. Competition among clones can be a major brake on the rate of resistance evolution because the onward transmission of resistant parasites acquired by either transmission or by *de novo* mutation can be stifled by competitors in the absence of drug treatment. Conversely, removal of drug-sensitive competitors by chemotherapy can greatly enhance the spread of parasites with high-level resistance. Thus, clone interactions in the presence and absence of drug treatment are major determinants of the useful lifespan of a drug [6], [36]–[42].

The development of evidence-based resistance management strategies requires an understanding of these clone-clone interactions within hosts [42]. Some analysis in human *Plasmodium* infections is possible [25], [43], but direct experimental investigations require that chemotherapy be denied to some individuals with malaria. Consequently, experimental work has focused on rodent models. There, competitive release has been demonstrated for resistant *P. chabaudi* parasites after prophylactic [28] and therapeutic [33]–[35] drug treatment. Competitive release can even lead to facilitation, where resistant parasites attain higher densities following the clearance of susceptible competitors than they would have achieved in single clone infections [33].

These rodent malaria studies have assumed that relative and absolute fitness (transmission success) of resistant parasites can be inferred from their density in gametocyte populations in peripheral blood sampled from the mouse tail vein. Transmission of human malaria to mosquitoes is generally positively related to gametocyte density within the host [44]–[46], and Hill et al. [47] observed that the extent of parasite multiplicity in infected people was reflected in the parasite diversity in *Anopheles* mosquitoes. A limited body of empirical evidence for *P. chabaudi* (e.g. [27], [28], [48]) and the lizard malaria, *P. mexicanum* [49], shows that gametocyte densities of co-infecting parasites in host blood correlate with transmission success of individual clones to mosquitoes (or sandflies in the lizard-malaria model). Here we determine whether this assumption holds true following chemotherapy. Clones of *P. falciparum* that survive drug treatment produce infectious gametocytes [13], but relative infectivity of resistant and sensitive parasites remains to be demonstrated. In *P. chabaudi*, drug treatment and competition independently affect determinants of infectivity (e.g. [50]–[53]) and there are situations where the clonal composition of parasite populations in mice is not well correlated with the genetic composition of parasites in mosquitoes fed on those hosts (e.g. [54]). Some authors have also suggested that variation in parasite transmission among mosquitoes fed on the same host at the same time might be in part due to aggregation of gametocytes in skin capillaries prior to ingestion by the mosquitoes [55], [56]. If so, the actual uptake of gametocytes from host capillaries by mosquitoes might differ from the gametocyte densities identified in tail-snip venous blood samples, particularly at low gametocyte densities.

Here we test whether transmission consequences of drug treatment as inferred from gametocyte densities do in fact play out in transmission in a *P. chabaudi* – *An. stephensi* model system. We deliberately initiated infections with a highly skewed ratio of sensitive to resistant parasites because we knew from previous work [57] that drug treatment would lead to substantial changes in clone frequencies. We determined the densities of resistant and sensitive parasites in the presence and absence of drug treatment at two life stages in both hosts: in the asexual and gametocyte populations in the mouse, and in the blood meal and oocysts in the mosquito. Parasite dynamics of mixed-clone infections in the vertebrate host were indeed reflected in the transmission success of individual clones.

## Methods

### Ethics Statement

The study was carried out in strict accordance with the recommendations in the guide for the Care and Use of Laboratory Animals of the National Institutes of Health. The protocol was approved by the Animal Care and Use Committee of the Pennsylvania State University (Permit Number: 35790).

### Parasites and Hosts

Two genetically distinct clones were used in the experiment: The pyrimethamine-sensitive clone AJ_5P_ (hereafter referred to as clone S) and the pyrimethamine-resistant clone AS_6P(pyr-1A)_ (hereafter referred to as clone R). Both clones were isolated from individual thicket rats and subsequently cloned as detailed by Beale et al. [58]. Clone R was made resistant by a single high-dose exposure to pyrimethamine [59].

Experimental murine hosts were 8 week old female C57Bl/6 laboratory mice (Charles River Laboratories). All mice were maintained at 26°C with a 12L:12D photoperiod, fed Laboratory Rodent Diet 5001 (LabDiet, PMI Nutrition International) and received drinking water supplemented with 0.05% para-amino benzoic acid to enhance parasite growth [60].

### Experimental Design, Drug Treatment and Mosquito Feeds

Twenty mice were each challenged with mixed infections of clone R (10^2^ parasites) and clone S (10^6^ parasites). We used these very unequal inocula to establish infections initially dominated by sensitive parasites; previous work [31], [57] had demonstrated that these starting conditions generate substantial competitive suppression of resistant parasites in the absence of chemotherapy, and substantial competitive release following drug treatment [31], [33]–[35]. Resistant parasites will often be rare in untreated infections in nature when, for instance, there is a competitive disadvantage associated with the costs of resistance [31], [33], [34], [40] or when resistance first arises *de novo*.

Inoculations were prepared from donor mice by diluting blood in 0.1 ml of calf serum solution (50% heat-inactivated calf serum, 50% Ringer’s solution [27 mM KCl, 27 mM CaCl2, and 150 mM NaCl] and 20 units of heparin per millilitre) and were introduced by intra-peritoneal (i.p.) injection. Ten mice were drug treated and ten were sham treated. Drug treatment was initiated on day six post-infection (PI), the time point at which pronounced anaemia and weight loss become apparent (see [33], [34]). Treatment consisted of 8 mg/kg pyrimethamine dissolved in dimethyl sulfoxide (DMSO), administered as a 50 µl i.p. injection on four successive days. Sham-treated mice received contemporaneous i.p. injections of 50 µl of DMSO without pyrimethamine.

To measure the transmission effects of treatment, *Anopheles stephensi* mosquitoes were allowed to feed on experimental mice over a week, starting two days after drug treatment finished. For the transmission experiments, twelve of the twenty mice were chosen at random among survivors (see results), six of whom had been drug-treated. In this feeding experiment, each mouse was used every second day, with half the mice allocated to even days (days 12, 14, 16, 18 PI) and half to the alternate days (13, 15, 17, 19 PI) cross-factored with drug treatment in a fully balanced experimental design. Thus, each mouse was offered to mosquitoes on four separate occasions over 8 days.

Approximately thirty mosquitoes (2–5 days old) were introduced into individual half-liter plastic cups via a slit in the mesh-covered top. The cups had roughened internal surfaces to facilitate mosquito resting and the mesh slit was plugged with cotton wool to prevent escape. An additional pad of cotton wool soaked in 5% glucose solution was placed on the surface of each cup’s mesh cover to enable feeding *ad. lib*. and the cups were kept in the incubator along with stock cages. Incubator conditions of 26°C, 70% relative humidity and a 12L:12D photoperiod were maintained throughout the course of the experiment. Glucose pads were removed from cups one hour before experimental blood feeds. Mice were anaesthetised immediately prior to blood feeds with a 0.05 ml i.p. injection of Ketamine (100 mg/kg)/Xylazine (10 mg/kg). Once unconscious, individual mice were laid upon the mesh covering of each cup and the mosquitoes allowed to blood feed for 30 minutes. Mice were then returned to their cages to recuperate, unfed mosquitoes were removed from cups and new glucose pads provided.

### Monitoring Infection Dynamics in Mice

Mice were sampled before noon on a daily basis between days five and 19 PI. A thin blood smear was made, morbidity monitored by recording mouse body mass (to the nearest 0.01 g) and 2 µl of blood taken via a tail snip to determine red blood cell (RBC) density using flow cytometry (Beckham Coulter). Additional 5 µl and 10 µl blood samples were collected for DNA and RNA extraction, respectively. Blood samples for DNA extraction were handled and extracted as detailed by Bell et al. [32]. Blood samples for RNA extraction were added to a chilled lysis mixture of 10 µl phosphate buffered saline (PBS: Ca^2+^/Mg^2+^ -free) and 20 µl Nucleic Acid Purification Lysis Solution (Applied Biosystems), the mixture immediately vortexed to allow complete lysis and the lysate kept on ice prior to storage at −80°C. Total RNA was extracted using the “RNA Blood-DNA” method, on the ABI Prism 6100 Nucleic Acid Prepstation, with an elution volume of 100 µl. RNA was immediately converted to cDNA using the High-Capacity cDNA Archive Kit (Applied Biosystems). Both RNA and cDNA were stored at −80°C.

Parasite densities were determined using clone-specific PCR primers and minor groove-binder (MGB) probes, targeting either the *P. chabaudi ama* gene (for quantification of total parasite densities [considered to equate to asexual parasite densities] from DNA [32]) or the *common gametocyte* gene (CG1, for quantification of gametocytes from cDNA; as [61]). Real-time quantitative PCRs were performed on an ABI Prism 7500 Fast System with an initial denaturation of 95°C for 2 min followed by 40 cycles of denaturation at 95°C for 3 sec and annealing/extension at 60°C for 30 sec. For the *ama* assay, 2 µl of DNA were included in a total PCR reaction volume of 25 µl with 1×PerfeCTa™ qPCR FastMix™ (Quanta Biosciences), forward and reverse primers at 300 nM and TaqMan® MGB probe (Applied Biosystems) at 200 nM. The CG1 cDNA assay incorporated 7 µl of cDNA (at a 1∶10 dilution) in a total reaction volume of 25 µl with 1×PerfeCTa™ qPCR FastMix™ (Quanta Biosciences), forward and reverse primers at 900 nM and TaqMan® MGB probe (Applied Biosystems) at 250 nM. Absolute quantification was based on a standard curve of serial dilutions of DNA and cDNA standards of known asexual parasite and gametocyte densities, respectively, determined beforehand by careful microscopy (validated by [32], [33], [61]–[64]).

### Monitoring Transmission to Mosquitoes

Immediately post-blood feed, two fully engorged mosquitoes were removed from each pot, anaesthetised with chloroform and placed individually into 1.5 ml microfuge tubes containing 500 µl of RNAlater™ (Qiagen), with care taken to ensure that mosquitoes were totally submerged. Tubes were stored at −80°C until RNA extraction. Mechanical disruption of mosquitoes was achieved with a TissueLyser (Qiagen) under the following conditions. Upon thawing, mosquitoes were removed from the RNAlater™ and placed individually into collection microtubes containing 600 µl of Buffer RLT from the RNeasy™ Protect Mini Kit (Qiagen), 0.25 g of sterile 0.2 mm zirconium beads (OPS Diagnostics, LLC) and 0.25 g of sterile 0.8 mm silica beads (OPS Diagnostics, LLC). Mosquitoes were then ground for 2 mins at 30 Hertz, with the microtubes repositioned within the TissueLyser every 30 s to ensure uniformity of disruption for all samples. RNA was then extracted using the RNeasy™ Protect Mini Kit (Qiagen) according to the manufacturer’s instructions and eluted in a volume of 100 µl. The RNA was handled and stored as detailed above for that obtained from mouse blood samples. Gametocyte densities present within mosquito blood-meals were quantified as described for mouse blood-derived cDNA.

Mosquitoes not sub-sampled for blood-meal analysis were kept within the incubator and fed glucose *ad lib.* until dissection at 9 days post-blood feed. Mid-guts were examined for intensity of infection (number of oocysts) using a compound microscope and infected guts placed individually into 30 µl of chilled PBS within 1.5 ml microtubes. Tubes were maintained on ice prior to storage at −80°C. DNA was extracted from individual mosquito mid-guts using the E.Z.N.A MicroElute Genomic DNA kit (Omega Bio-Tek) as per manufacturer’s instructions, eluted in a total volume of 20 µl and stored at −80°C. Clone densities present within oocysts on mid-guts were determined as for DNA samples obtained from mouse blood samples.

### Statistical Analyses

To summarize asexual and gametocyte parasite densities through time, the geometric mean densities were calculated for clone R, clone S or both (total densities) for each mouse for the entire infection course (d5 – d19 PI) and for feed days (either days 12, 14, 16, 18 or days 13, 15, 17 and 19 PI, dependent upon mouse). General linear modeling (MINITAB v. 14) was used to compare the effect of drugs (treated or untreated) on parasite densities. The relationship between gametocyte density and the probability of mosquito infectivity for the two clones was studied using logistic regression. Logistic regression was performed in the statistical software environment R (version 2.11.1 [65]), using a generalized linear model with a binomial distribution and logit link function. The predicted infectivity and 90% prediction interval (i.e., 90% probability that future observations will fall within these bounds) were estimated from the fit model. Since regression parameters (*a* and *b*) were estimated on the logit scale, predicted values were obtained using the logit transformation
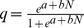
(1)where *a* and *b* are parameters from the linear regression *π* = *ax+b* on the logit scale.

## Results

### Mouse Morbidity

Four sham-treated mice had to be euthanized before the commencement of mosquito feeds in accordance with animal care guidelines. All drug-treated mice (n = 10) remained outwardly healthy with significantly less weight loss (total weight loss: F_1,15_ = 57.4, p<0.001) and anaemia (minimum RBC density: F_1,15_ = 53.4, p<0.001) than non-drug treated mice (n = 6) during the monitoring period (days 5–19 PI).

### Parasite Performance Prior to Drug Treatment

**Figure 1:**
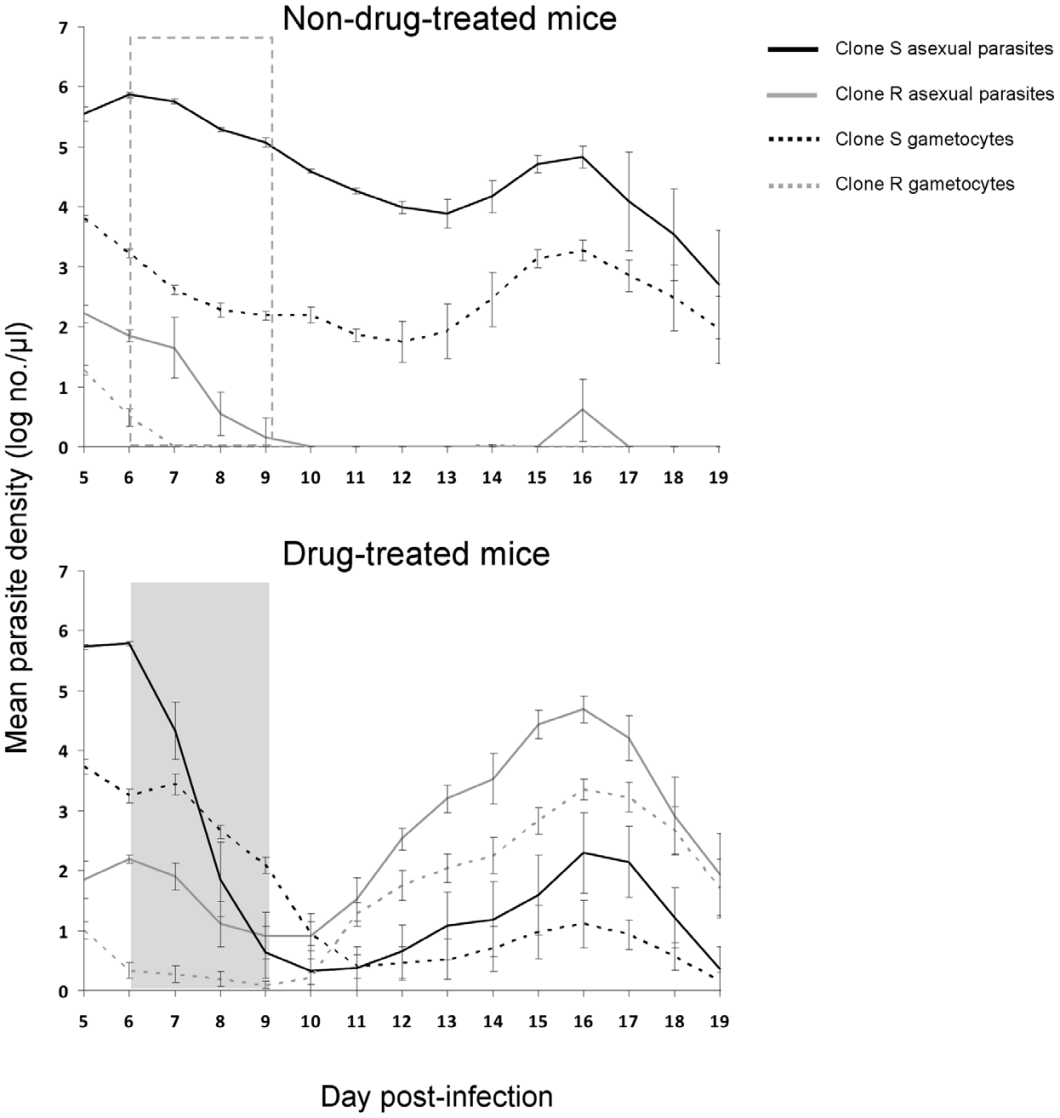
Densities of asexual parasites (solid lines) and gametocytes (dotted lines) of drug-sensitive (clone S, black) and drug-resistant (clone R, gray) parasites within sham-treated (top panel) and drug-treated (bottom panel) mice. Dashed and shaded boxes show days of sham or drug treatment. Data are log-transformed geometric means (± S.E) of a maximum of 10 mice (number of untreated mice [top panel] reduced to six by day 12 due to morbidity-driven euthanasia).

**Figure 2:**
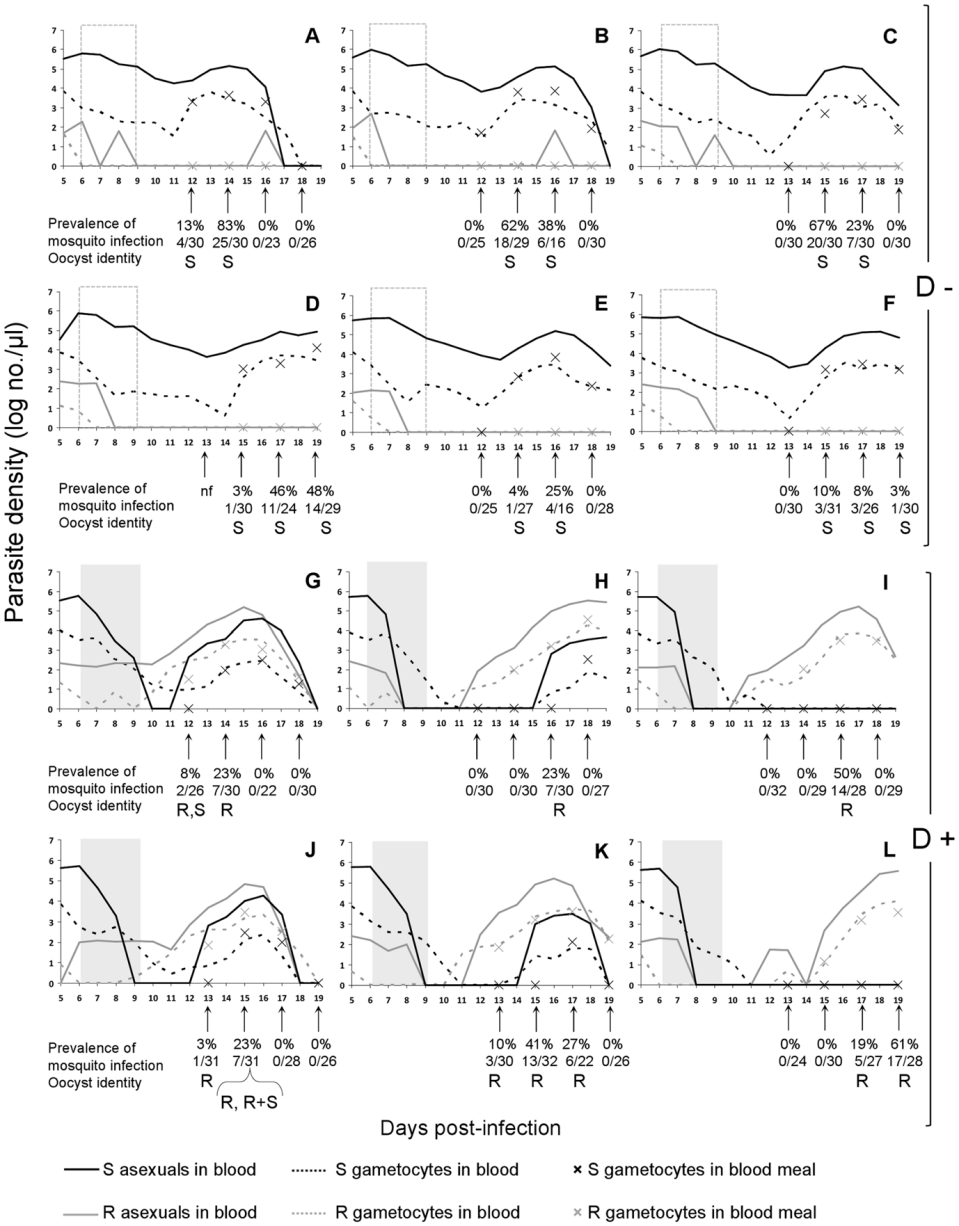
Asexual and gametocyte densities of clone S and clone R within the 12 randomly selected mice used for mosquito blood feeds (see methods), gametocyte densities within blood-meals (denoted by crosses), prevalence of mosquito infection (percentage and numbers of infected mosquitoes at each feed) and identity of oocysts present on mid-guts 9 days post-blood feed (S: clone S genotype; R: clone R genotype; R+S: both clone genotypes). Panels A-F, non-drug-treated mice (D−); panels G–L, drug-treated mice (D+); nf: no blood feed performed due to mouse morbidity. Dotted and grey blocks show period of sham or drug treatment.

Before drug treatment began on day 6 PI, there were no significant differences in parasite densities within hosts of each treatment group (Figures 1 and 2; resistant asexuals: F_1,19_ = 1.1, p = 0.31; resistant gametocytes: F_1,19_ = 0.7, p = 0.42; susceptible asexuals: F_1,19_ = 2.6, p = 0.13; susceptible gametocytes: F_1,19_ = 0.03, p = 0.86). The 10,000-fold difference in the densities of sensitive and resistant parasites we created at the beginning was maintained through to the start of drug treatment at day 6 p.i. (Figure 1– both panels; Figure 2), at which point there were only 179.5 (±26.8 [s.e.m]) resistant asexual parasites and 3.6 (±1.02) resistant gametocytes per microliter of mouse blood.

### Fate of Resistant Parasites in Treated and Untreated Mice

In the absence of drug treatment, the infection was dominated by the susceptible parasites (Figure 1– top panel; Figure 2– panels A–F). Indeed, in four of six untreated mice, resistant asexual parasites were excluded from the infection (below stochastic detection levels) by day 10 PI. Gametocytes from the resistant clone were detected on only two days at most in untreated mice, and never after day 7 PI. Thus, in the absence of drug treatment, the resistant clone had negligible transmission potential.

**Figure 3:**
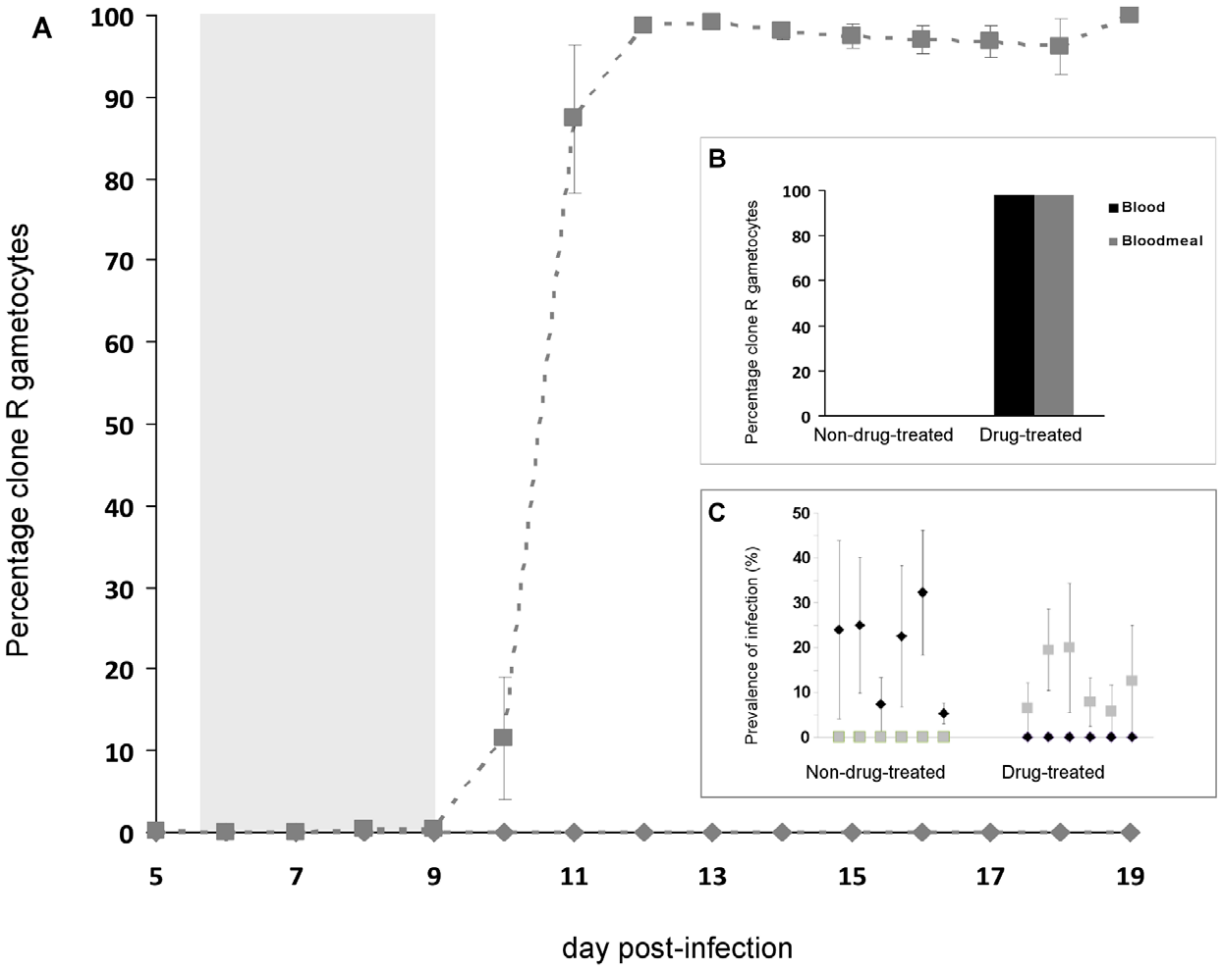
Transmission parameters. **A**. Frequency of gametocytes of the resistant clone present in the blood of sham-treated (diamonds) and drug-treated (squares) mice used for mosquito blood feeds. Shaded area indicates timing of treatment. Each data point represents the mean (±1 S.E.M) from six mice. **B**. Percentage of clone R gametocytes in mouse blood at time of mosquito blood feeds (dark bars) and in mosquito blood-meals fed on those mice (gray bars), for non-drug-treated and drug-treated mice. There were no gametocytes from the resistant clone in untreated mice. **C**. Prevalence of infection with each clone in mosquitoes fed on each of the six treated and six sham-treated mice. Plotted points are the mean (± SEM) for each mouse across all four feed days, with *c.*30 mosquitoes per feed. Diamonds, S alleles; squares, R alleles. (2 of 83 infections corresponding to either S or S+R alleles are not included).

At the start of drug treatment, resistant parasites constituted less than 0.05% of the parasite and gametocyte populations. After treatment, the population of resistant parasites rapidly expanded (Figure 1– bottom panel, Figure 2– panels G–L), so that the resistant clone became the numerically dominant two days after the cessation of drug treatment (day 11 PI). A day later, the resistant clone made up more than 95% of the parasite population (Figure 3). Drug treatment completely cleared susceptible parasites in only two of the six mice used for mosquito blood feeds (Figure 2– panels I & L). In the other four mice, susceptible parasites were undetectable for 2 to 8 days before they recrudesced to varying degrees (Figure 2– panels G, H, J & K).

### Total Parasite Burdens

In the absence of drug treatment, parasite populations were almost exclusively clone S, whereas drug-treated populations were dominated by the resistant clone (Figures 1 and 2). For mice used in the mosquito feeds, drug treatment reduced total asexual parasites densities across the monitoring period (sum of R & S clones; geometric means across day 5–19 PI; F_1,11_ = 38.4, P<0.001).

Total gametocyte densities were unaffected by drug treatment (F_1,11_ = 0.58, P = 0.46): drug treatment simply replaced drug-sensitive gametocytes with drug-resistant gametocytes (Figures 1 and 2).

### Transmission to Mosquitoes: Blood-meals and Oocysts

Gametocyte densities in mosquito blood-meals over time, and the prevalence of infection in mosquitoes(% with oocysts on the midgut) are shown in Figure 2. Direct quantitative comparisons between gametocytes present in a micro-liter of mouse blood and within a mosquito blood-meal taken from that mouse must take into consideration that different RNA extraction protocols were employed for each. Indeed, gametocytes were typically not quantifiable within the blood-meal when the gametocyte density in mouse blood was less than about 30 gametocytes/µl (Figure 2– panels C, E–L).

In the absence of drug treatment, no clone R gametocytes were detectable in mouse blood at the time of mosquito feeds and so, unsurprisingly, no resistant gametocytes were identified in mosquito blood-meals (Figures 3A & 3B) and none of the oocysts which subsequently developed contained resistant parasites (Figure 2– panels A, B, C, D, E, F). Drug treatment reversed this, with resistant parasites dominating in treated mice at the time of mosquito blood feeds (98.8%), in the blood meals of mosquitoes feeding on those mice (98.0%), and in the oocysts which subsequently developed (98.6% of 139 oocysts, 99.6% of the genomes [ =  sporozoites]) (Figures 3A, 3B & 2– panels G, H, I, J, K, L).

Drug treatment did not have an impact on infectiousness of the mice (Fig 3C; F_1,11_ = 2.1, p = 0.18), with similar proportions of mosquitoes becoming infected from non-drug-treated (19.4%, ± S.E: 4.4; range of 5.3 to 32.3%) and drug-treated mice (12%, ± S.E: 2.6; range of 5.8 to 20%).

### Relationship Between Gametocyte Densities in Mouse Blood and Prevalence of Infection in Mosquitoes

**Figure 4:**
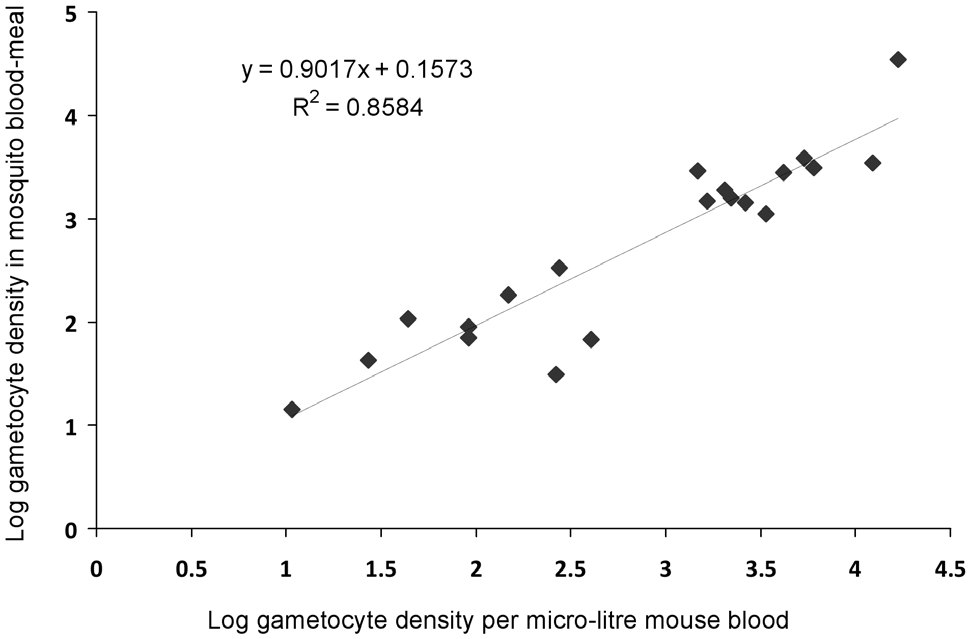
Relationship between clone R gametocyte densities in mice at the time of a blood feed and in mosquito blood-meals fed on those mice.

The densities of R gametocytes in mouse blood on feed days and in the blood-meals of mosquitoes that fed on that mouse were highly correlated across more than 4 orders of magnitude, with a regression slope nearing 1 and an intercept not distinguishable from zero (Figure 4; F = 109.1, p<0.001, slope = 0.902±0.09, intercept = 0.16±0.26). This means that any clumping of gametocytes in host capillary beds [55], [56] is not affecting the efficiency of transmission of gametocytes to mosquitoes.

**Figure 5:**
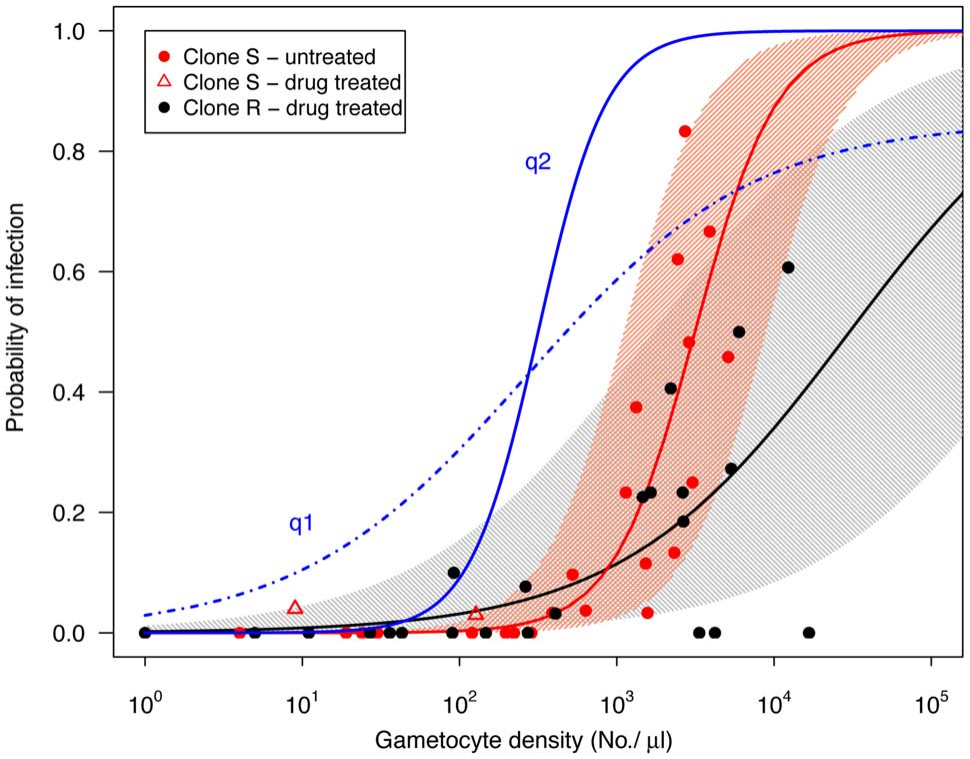
Relationship between gametocyte densities in mice at the time of a blood feed and the subsequent prevalence of infection in mosquitoes fed on those mice for untreated infections with transmission of clone S (red points) and for drug-treated infections with transmission of clone R (black points). The thick red and black lines show the predicted probability of mosquito infection based on logistic regression (eq 1), and the shaded areas show the 90% prediction intervals (note that these are not confidence intervals, see text for details). Two oocysts consisting of clone S were observed in drug treated infections (open red triangles), these were not included in the model (see text). Blue lines are gametocyte density-infectivity functions (of the form *q* = α*N* ^β^/[1+ γ*N* ^β^]) estimated from *P. falciparum* data compiled by Carter and Graves [66] and Barnes & White [67]: q1 (dot-dashed blue line;α = 0.03, β = 0.6, α/γ = 0.85) and q2 (solid blue line; α = 1·10−5, β = 2, α/γ = 1) as presented by Huijben et al. [34].

In the absence of drug treatment, only clone S was transmitted to mosquitoes. Successful formation of oocysts was observed only when gametocyte densities exceeded 10^2.5^/µl of mouse blood (Figure 5). Prevalence of infection rose sharply with increasing gametocyte densities, withevery mouse exceeding this threshold infectious to mosquitoes. Following drug treatment, successful transmission was almostentirely due to resistant parasites, although one mosquito harboured a single oocyst with just the clone S genotype (Figure 2– panel G) and another mosquito bore an oocyst that had apparently resulted from cross-fertilization (contained both clone genotypes; Figure 2– panel J). Transmission of the resistant clone after drug treatment occasionally occurred at lower gametocyte densities than was observed for sensitive clone transmission, but also sometimes failed at densities greater than 10^3.5^ per micro-liter mouse blood (Figure 5). Interestingly, the two oocysts with clone S alleles that established from drug-treated mice did so when densities of sensitive gametocytes in the blood were lower than apparently necessary for transmission in the absence of drug treatment (Figure 5).

The relationship between gametocyte density and probability of mosquito infection is steeper in untreated mixed infections with clone S transmission than drug-treated infections with clone R transmission (Figure 5; *clone*: χ^2^(1, N = 45) = 195, p<0.001, *clone* x *gametocyte density*: χ^2^(1, N = 45) = 163, p<0.001). The flatter slope of the latter can be partly explained by three time points from three different mice (feeds from one mouse on day 16 and two mice on day 18) when R gametocyte densities over the transmission threshold did not result in infections (Figure 5). However, removing these three data points still revealed a significant slope difference (*clone* x *gametocyte density*: χ^2^(1, N = 42) = 87, p<0.001). The gametocyte density-infectivity relation is given by
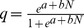
(2)where *q* is the probability a mosquito becomes infected, N is the log_10_ gametocyte density and parameters *a* = −6.37 (±1.28 SE) and *b* = 1.42 (±0.37 SE) for clone R and *a* = −12.69 (±3.03 SE) and *b* = 3.60 (±0.90 SE) for clone S. These infectivity functions have a higher threshold density than those for *P. falciparum* (Figure 5, [34], [66], [67]). The parameterized density-infection function can be used to estimate infection thresholds. For example, if we consider a minimum infection probability of 5%, then we predict that clone R will require densities in the range of 10^1.2^ to 10^3.7^ gametocytes per µl of blood, and a range of 10^2.2^–10^3.2^ for clone S to transmit the infection.

## Discussion

As observed in previous studies [28], [33], [34], we found that removal of drug-sensitive parasites by drug treatment led to profound expansion the population of resistant parasites. We now show experimentally that this release dramatically affects transmission to mosquitoes, as was assumed in those earlier studies. In sham-treated mice, resistant parasites were suppressed to such an extent that transmissible gametocytes were undetectable in peripheral mouse blood from day 10 post-infection, and in the blood-meals of mosquitoes subsequently fed on these mice. Moreover, resistant parasites did not contribute to any oocysts generated by those infections. In contrast, the clearance of sensitive parasites by chemotherapy led to resistant gametocyte densities rising from barely detectable to approximately 90% of transmissible parasites 48 hours after the cessation of treatment (Figure 3A). Consequently, nearly all of the parasites (sporozoites) in oocysts established on mosquito mid-guts (99.6%) were from the resistant genotype (Figure 2, panels G, H, I, J, K, L).

The density of resistant parasites within the gametocyte population in mouse blood correlated closely with densities recorded in mosquito blood-meals (Figure 4). This indicates that counts obtained from tail-snip venous blood are an accurate representation of those acquired by the mosquito: there is no evidence that any gametocytes clumping in host capillaries resulted in a difference in the genetic composition of the gametocyte populations taken up by individual mosquitoes.

The gametocyte density-infectivity relation curves (Figure 5) differed between clones, with a steeper increase in infectivity per gametocyte density for clone S (Figure 5). This difference could be due to a genetic difference between the clones, which may or may not be related to resistance *per se*. Alternatively, the shallower slope could be a result of drug treatment (the clone R data are all from treated mice, and the clone S from untreated mice), or it might reflect differences in the infectivity of gametocytes originating from primary (clone R) or secondary (clone S) parasite peaks. Experiments analogous to those performed here could be used to investigate further the impact of parasite genetics, drug treatment and immunity on these dose-response curves.

Gametocyte density-infectivity relationships are expected to be s-shaped, with a density threshold below which negligible transmission can occur, an initially accelerating curve as the probability of gametes of both sexes finding each other in a mosquito blood meal increases, then an approximately linear phase during which an increasing number of gametocytes increases the likelihood of establishing an infection within the mosquito, and then finally saturation at some upper-bound prevalence. This prevalence at saturation can be lower than 100% if, for example, there are refractory mosquitoes in the population (e.g. [67]). Extrapolating from the model fitted to our *P. chabaudi* data, saturation is expected to occur at densities over 10^4^ gametocytes/µl, densities that are rarely attained in this host-parasite system.

The WHO recommends that once more than 10% of patients are failing to respond to treatment with a particular drug, that drug be withdrawn from front-line use by national authorities [4]. One way to prevent the spread of parasites with resistance levels high enough to render a drug insufficiently efficacious for clinical use is to preemptively kill sensitive or semi-resistant parasites with drugs, since dead parasites cannot mutate to the drug-threatening high-level resistance. This is the resistance management justification for designing treatment regimens aimed at removing all parasites as fast as possible from a patient [4]. This strategy can be an important weapon in the fight against drug resistance, but our data clearly demonstrate the unavoidable downside of this strategy [42]: it confers exceedingly strong evolutionary advantages on any high-level resistance that is already present in an infection. The resistant clone used in this experiment has previously been shown to be unaffected by the doses of pyrimethamine used here [33]–[35] and so it has the high-level resistance that resistance management strategies are trying to prevent from spreading. In our experiments, co-infection with the sensitive clone prevented the resistant clone from transmitting to mosquitoes. Chemotherapy eliminated the sensitive parasites and handed essentially all the transmission to the resistant parasites. Clearly, mice are not men (as discussed in this context by Wargo et al. [33]), but it seems plausible that competitive suppression will also occur in human infections. If so, the process of competitive release will be a major determinant of the rate of spread of high-level resistance in a population [38]. Note that it affects both the probability a patient will transmit resistant parasites acquired from others as well as the probability that any *de novo* mutations to high level resistance are able to reach transmissible frequencies and escape from a patient in the first place. Such processes may occur not only following therapeutic drug use, but also following prophylactic chemotherapy such as intermittent preventative therapy (IPT) in pregnant women, infants or children [25], [42] and will likely be dependent on the ecological factors such as frequencies at the time of treatment and the presence of other clones at the time of infection [30], [35], [57].

Thus, chemotherapy is a double-edged sword for resistance management [42]. It can, by killing parasites, control the probability that *de novo* mutants will occur in a patient in the first place, but only at the cost of imposing very strong selection for any that are already there. Chemotherapeutic regimens designed to remove all parasites as fast as possible can confer substantial benefits (e.g. clinical gains, or minimizing the number of parasites which are alive to mutate), but they will have the highly undesirable consequence of maximally spreading any high-level resistant parasite that is present. A better understanding of clone-clone interactions, and of the effect of different drugs and treatment regimens on those interactions, may help identify new ways to improve patient health while limiting the transmission advantages chemotherapy confers on resistance parasites. Meanwhile, treated patients should be urged to use bednets in the period after treatment to reduce the chances they will spread resistant parasites to others.
